# Influence of peer support on HIV/STI prevention and safety amongst international migrant sex workers: A qualitative study at the Mexico-Guatemala border

**DOI:** 10.1371/journal.pone.0190787

**Published:** 2018-01-05

**Authors:** Belen Febres-Cordero, Kimberly C. Brouwer, Teresita Rocha-Jimenez, Carmen Fernandez-Casanueva, Sonia Morales-Miranda, Shira M. Goldenberg

**Affiliations:** 1 Gender and Sexual Health Initiative, British Columbia Centre for Excellence in HIV/AIDS, Vancouver, British Columbia, Canada; 2 Division of Global Public Health, University of California, La Jolla, California, United States of America; 3 Centro de Investigaciones y Estudios Superiores en Antropología Social, Chiapas, Mexico; 4 HIV Unit, Universidad del Valle, Guatemala City, Guatemala; David Geffen School of Medicine at UCLA, UNITED STATES

## Abstract

**Background:**

Migrant women engaged in precarious employment, such as sex work, frequently face pronounced social isolation alongside other barriers to health and human rights. Although peer support has been identified as a critical HIV and violence prevention intervention for sex workers, little is known about access to peer support or its role in shaping health and social outcomes for migrant sex workers. This article analyses the role of peer support in shaping vulnerability and resilience related to HIV/STI prevention and violence among international migrant sex workers at the Mexico-Guatemala border.

**Methods:**

This qualitative study is based on 31 semi-structured interviews conducted with international migrant sex workers in the Mexico-Guatemala border communities of Tapachula, Mexico and Tecún Umán and Quetzaltenango, Guatemala.

**Results:**

Peer support was found to be critical for reducing social isolation; improving access to HIV/STI knowledge, prevention and resources; and mitigating workplace violence, particularly at the initial stages of migration and sex work. Peer support was especially critical for countering social isolation, and peers represented a valuable source of HIV/STI prevention knowledge and resources (e.g., condoms), as well as essential safety supports in the workplace. However, challenges to accessing peer support were noted, including difficulties establishing long-lasting relationships and other forms of social participation due to frequent mobility, as well as tensions among peers within some work environments. Variations in access to peer support related to country of work, work environment, sex work and migration stage, and sex work experience were also identified.

**Conclusions:**

Results indicate that peer-led and community empowerment interventions represent a promising strategy for promoting the health, safety and human rights of migrant sex workers. Tailored community empowerment interventions addressing the unique migration-related contexts and challenges faced by migrant sex workers should be a focus of future community-based research, alongside promotion of broader structural changes.

## Introduction

Women comprise nearly half of international migrants worldwide and in Latin America and the Caribbean, a region representing 15 per cent (37 million) of the global international migrant stock in 2015 (244 million) [1]. Known as the ‘gateway to the Americas’, the Mexico-Guatemala border is a crucial migration site. Approximately half a million undocumented migrants are reported to cross the Mexico-Guatemala border annually, most of whom are international migrants from Central America aiming to reside in the United States or Canada [2–4]. However, evidence regarding the health and safety of women who migrate internationally within this highly mobile border context remains extremely scant [5–7]. Although most research on the health of migrant workers has focused on males, emerging work highlights the health and social inequalities faced by female migrants, including socioeconomic marginalization, barriers to healthcare, and social isolation [5,8,9]. Social isolation, gender inequities, racialization and discrimination, and legal barriers related to migration status in destination communities are among the barriers which commonly limit migrant women’s access to conventional labour markets, resulting in their over-representation in precarious and under-regulated forms of work, including the sex industry [6,10]. Migrant women engaged in sex work often face intersecting health-related challenges,–such as violence, elevated risk of HIV/STI infection, abuse/extortion from authorities and social isolation, all of which operate at multiple levels [5,9,11].

This study drew on conceptualizations of health as shaped by factors at multiple levels of influence, including prior theoretical frameworks of structural determinants of sex workers’ health [12]. Such frameworks conceptualize peer support as interacting with factors at several macrostructural, community, social, physical, policy, and economic levels to shape HIV and sexual health amongst sex workers [12]. Our study was also more broadly informed by the Socioecological Model of health [11], which recognizes five main overlapping levels of influence on health outcomes: intrapersonal factors (e.g., individual characteristics such as knowledge, beliefs, and self-concept), interpersonal processes and primary groups (e.g., individual’s social environment such as family, friends, and peers), institutional or organizational factors (e.g., workplaces and other social institutions with formal or informal policies and structures), community factors (e.g., relationships among organizations and institutions), and public policies (e.g., public policies or regulations concerning health). These categories enable an examination of the overlapping factors determining the health and wellbeing of migrants and other social groups operating at these different levels [11,13,14].

Previous research has identified social isolation as one of the key interpersonal determinants of migrant health. Defined as the “state in which the individual lacks a sense of belonging socially, lacks engagement with others, has minimal number of social contacts and they are deficient in fulfilling and quality relationships”[15], social isolation is considered a risk factor among different population groups for morbidity and mortality from diverse causes including cardiovascular disease, accidents and suicide [16–20]. Social isolation has been especially implicated in mental health disorders among the general population [21,22], older adults [23,24] and migrants [25,26], and in violence and HIV/STI risk among migrants [8,27–30]. Within destination settings, migrants–particularly unaccompanied women–often face difficulties establishing and accessing social networks and support, due to intersecting factors including displacement, lack of kinship networks, concerns related to legal migration status, and experiences of stigma and discrimination [31,32]. Previous research has found that social isolation is often present in the lives of marginalized women prior to migration and / or sex work entry, and it is frequently a driving factor for both, alongside poverty and family needs [5,6]. Social isolation may be further compounded for this group by the criminalized and highly stigmatized nature of sex work [3,32]. Studies conducted in diverse contexts including Central America have demonstrated that macrostructural factors, such as punitive public health regulations and laws, can further increase the criminalization and stigmatization faced by migrant sex workers, and can elevate the risk of social isolation among this population [7,33].

Limited research has investigated social isolation and support among migrant sex workers. Although migrant sex workers often face extensive social isolation and exclusion due to separation from support networks, criminalization, and punitive sex work regulations, very little is known about the ways in which these experiences relate to access to healthcare services or augment the hazard of experiencing violence within the workplace and harassment from authorities [12]. Prior research with the general population of sex workers, however, has shown that social isolation can significantly undermine safer working conditions and sex workers’ ability to negotiate condom use and HIV/STI prevention [3,34].

Conversely, social and peer support–defined as ‘the emotional, instrumental, and financial aid that people with similar life experiences offer to each other’ [35,36]–has been shown to be important for mitigating health and social inequities faced by marginalized and socially isolated populations. Emerging literature has documented positive health outcomes (e.g., fewer unprotected sex acts, reductions in HIV and STI infections) resulting from community empowerment-based approaches and interventions focusing on mobilizing communities and improving social support and social cohesion among sex workers across diverse settings including India, Brazil, Mexico, the Dominican Republic and Canada [34,37–41]. Such interventions are particularly relevant in contexts such as Central America and other low and middle-income settings where sex workers’ increased risk for HIV is characterized by social and structural constraints including criminalization and penalization of sex work; and intersecting social stigma, discrimination, and violence related to occupation, socioeconomic position, gender, and migration status This data is crucial in communities within this region characterized by intense mobility, such as the Mexico-Guatemala border [3,7,33,39,42]. However, the majority of this research has been conducted with non-migrant women and little is known about access to peer support or its role in shaping health and social outcomes within the context of international migration. Peer support has been shown to be critical for mitigating stigma and health inequities facing sex workers generally[12,34,41,43]. Yet, migrant sex workers may face unique challenges accessing peer support and engaging in community empowerment initiatives. Research on this topic with migrant women remains critically needed given the fact that concerns regarding legal status, frequent mobility, separation from previous networks of support, and stigma associated with both migration and sex work can pose unique challenges for migrant sex workers [5,44] that may require unique intervention approaches.

Given gaps in evidence regarding peer support in relation to the health of migrant sex workers, we undertook this qualitative study situated in the Mexico-Guatemala region to analyze the role of peer support in shaping vulnerability and resilience related to HIV/STI prevention and violence among international migrant sex workers. In Guatemala, the CA-4 free transit agreement allows migrants from neighboring countries (El Salvador, Honduras, Nicaragua) to visit for up to 90 days, but does not provide work authorization [45]. Immigration laws in Mexico do not permit Central Americans to visit or work in the country without a specific migration form or a regional visitor card, which is only applicable for people from Guatemala and Belize. Visitors and workers who do not have these permits are subject to deportation in both countries [46,47].

Migrant women in this region report entering the sex industry for a variety of reasons, with some migrating with the intention of engaging in sex work, and the majority reporting having entered into the industry following migration as the result of social and structural influences including economic hardship following arrival, subsistence needs, and limited access to other employment opportunities offering comparable earnings [6,48].

As in the rest of the world, sex workers on both sides of the border face disproportionately high rates of HIV and STI [7,8,32,33,49]. In Mexico and Guatemala, sex work is tolerated within certain indoor venues located at specific zones under policies designed to protect public health [7,50]. Previous research conducted in this region has found that the public health regulations in force in both countries that require sex workers in indoor venues to undergo periodical HIV/STI testing at municipal clinics, to maintain a health permit to engage in sex work, and to demonstrate compliance at the request of health authorities can facilitate access to testing among sex workers within these settings [9,29,51–55]. In Guatemala, municipal HIV/STI testing and health permits are provided free-of-charge through centrally-located community health clinics, while in Mexico sex workers have to pay for them and arrive to usually isolated clinics by their own means [51,56,57]. As authorities in both countries primarily require health permit possession in formal indoor venues, women working within informal venues and public street-based settings are much less likely to maintain such permits and face restricted access to regular HIV/STI testing [7,58,59].

Through our analysis of peer support among international migrant sex workers within these settings we aim to strengthen understandings of the potential role of peer-based interventions in consolidating resilience related to social isolation, HIV/ STI prevention, and violence faced by international migrant women engaged in the sex industry, while considering their possible relation to other individual, community, and contextual factors shaping migrant sex workers’ health and wellbeing.

## Methods

### Study setting

This qualitative study was conducted in three communities at the Mexico-Guatemala border region: Tapachula (Mexico), and Tecún Umán and Quetzaltenango (Guatemala). Study locations were selected based on their significance for frequent mobility and internal, cross-border, and intercontinental migration patterns [2–4]. Frequently driven by structural factors (e.g. gender-based violence, economic difficulties) [6,9], female migrants within this region are greatly overrepresented within the sex industry locally, with sources suggesting that over two-thirds of sex workers in our study sites are Central American migrants, most of whom arrive primarily from Honduras, El Salvador and Nicaragua [9,55,60].

Sex work in this site takes place both in formal establishments (e.g., bars or nightclubs) and informal venues (e.g., hotels, motels, private rooms, trucks or trailers). Previous research in this location has found that migrant sex workers’ health is greatly shaped by workplace characteristics given that within formal indoor spaces, some manager policies and practices can increase access to condoms and to HIV and STI information. In addition, protection from physical, sexual, verbal and psychological violence as well as from drug and alcohol use in these settings tends to be more present than in informal venues, where women often experience limited access to workplace support due to the more isolated nature of these environments [6,7,52,53].

### Ethics statement

The study was approved by institutional review boards at the University of California, San Diego; the Universidad del Valle de Guatemala (UVG); the Guatemalan Ministry of Public Health and Social Assistance; and El Colegio de la Frontera Sur (ECOSUR) and Centro Nacional para la Prevención y el Control del VIH/SIDA (CENSIDA) in Mexico. The informed consent process ensured that participation in this study was completely voluntarily and participants were informed that they could choose to stop or terminate the session at any moment, and that they could withdraw from the study at any time. Strict protocols were undertaken to protect participant confidentiality and privacy, including identifying participants by pseudonyms only, removal of personal identifiers from the transcripts, and careful measures to protect all study-related data.

### Data collection

Data for this analysis was drawn from field research conducted from November 2012 to January 2014 by a team of U.S., Canadian and Mexican researchers in partnership with local community-based HIV, sex work, and women’s organizations. All individuals received a detailed explanation of the study and were guided through the informed consent process by trained interviewers prior to participating; written informed consent was provided by all women.

As previously described [6], eligible participants were: female; internal migrants (i.e., currently living in a different city, town, or State than where they were born) or international migrants (i.e., those currently living outside their country of origin) [61]; aged 18 years old or older; self-reported having exchanged sex for money in the last month; and were able to provide informed consent. Participants were recruited at diverse indoor and outdoor sex work venues through unobtrusive invitations during community-led outreach.

Participants were selected using a purposive sampling approach aiming to gather diverse experiences related to migration and sex work, such as recent vs. long-term migrants, and formal vs. informal work settings. A total of 52 migrant female sex workers participated in the study. Given the focus of this analysis on social and structural experiences related to peer support and international migration (e.g., social isolation, circular mobility, harassment from authorities, prolonged durations away from home countries), the analysis was restricted to 31 international migrant women [61] engaged in sex work in the Mexico-Guatemala border communities of Tapachula (n = 13), Mexico and Tecún Umán (n = 11) and Quetzaltenango (n = 7), Guatemala.

Trained female staff from community partner organizations and from the University of California, San Diego (UCSD) conducted individual in-depth interviews in private storefront offices or at a confidential location of participants’ choosing (e.g., home, workplace). Interviews were audiotaped with participants’ consent, and lasted 1–2 hours. Aiming to collect women’s experiences regarding health, safety, migration, and sex work, the interviews loosely followed a semi-structured guide addressing topics including recent and lifetime migration experiences, sex work entry, HIV/STI risks, violence, access to healthcare, interactions with authorities, working conditions, social isolation, peer support, and recommended strategies for improving sex workers’ health and safety. The guide was iteratively revised by the research team as the data analysis and the collection process progressed, and as new themes and greater understanding emerged. Following the interview, participants completed a brief socio-demographic survey gathering information including age, age of sex work entry, country of origin, migration status, work environment, and duration of migration.

Data collection was also complemented by periodic modified ethnographic fieldwork over the study period (14 months), including visits to different sex work venues (e.g., bars, cantinas, street corners), health and social service provision agencies (e.g., municipal clinics, migration agencies) and informal conversations with health providers and community members, which contributed to our broader understanding of the context of migration, sex work, community organization, and access to health care in the study sites.

### Data analysis

All interviews were transcribed and translated by trained bilingual staff at UCSD. To assure confidentiality, personal identifiers were removed and substituted by pseudonyms. Data was coded using the software NVivo 11 (QSR, Australia). Coding was based on principles of inductive analysis to identify and compare common themes and patterns across participants [62].

The data was initially organized and coded using open coding to identify major themes emerging in the transcripts, including migration patterns and drivers, reasons for sex work entry, perceived HIV/ STI risks and prevention strategies, and experiences of violence. As peer support and social isolation arose as important, inter-related themes that pertained to international migrant sex workers’ health and safety within the initial codes, more detailed analytical codes were developed focusing on accounts of social and peer support, tensions among sex workers, and social isolation among international migrant sex workers.

### Participant characteristics

Of 31 international migrant sex workers participating in this study, 18 were interviewed in Guatemala and 13 in Mexico. The mean age was 33 years old. Primary countries of origin were Honduras (n = 11), El Salvador (n = 9), Guatemala (n = 8), and Nicaragua (n = 3), and the average duration of time spent in their current city was 6 years. The majority of participants (n = 20) had been deported from Mexico, Guatemala or the United States at least once. The mean age of sex work entry was 22 years old, and most women (n = 16) serviced clients in informal venues such as hotels or rented rooms, while others did so at bars or cantinas (n = 12), trailers or trucks (n = 2), or their home (n = 1) (Table 1). In light of increasing crackdowns and closures of numerous bars and cantinas in Tapachula during the study [63,64] all women recruited in Mexico worked in informal settings (n = 13), whereas the majority of those recruited in Guatemala worked in more formal indoor venues (n = 10).

**Table 1:** Socio-demographic characteristics of international migrant sex workers (N = 31) in the Mexico-Guatemala border.

Measure	*N*
**Country of interview**	
Tecún Umán or Quetzaltenango, Guatemala	18
Tapachula, Mexico	13
**Age, mean (min, max)**	33 (20, 47)
**Country of origin**	
Honduras	11
El Salvador	9
Guatemala	8
Nicaragua	3
**Years in current city, mean (min, max)**	6.8 (0.02, 30)
**Ever deported**	20
**Age of sex work entry, mean (min, max)**	22 (13, 42)
**Place of service**	
Hotel/motel/rented room	16
Bar/Cantina	12
Trailer/Truck	2
Own home	1

## Results

Participants faced intersecting challenges related to social isolation; limited access to HIV/STI knowledge, prevention and resources; and workplace violence. International migrant sex workers put into practice peer support mechanisms to reduce these difficulties throughout their migration process and their work. Peer support was found to be an important mitigating factor for addressing social isolation and limited access to HIV and STI knowledge and prevention; and for decreasing and responding to vulnerability to sexual, physical, verbal and psychological violence from clients, venue managers and authorities within the workplace. Variations and limitations in access to peer support related to current country of work (Mexico vs. Guatemala), work environments (formal vs. informal settings), migration stage (e.g., recent vs. long-term migrant), and sex work experience were also identified.

### Social isolation and the mitigating influence of peer support

International migrant sex workers described facing pervasive social isolation across different stages of migration, including pre-departure, during transit, and upon arrival, which was frequently linked to stigmatizing and isolating experiences encountered within the context of being a foreigner as well as a sex worker. Participants often reported limited access to social and economic support in their country of origin, which was frequently attributed to experiences of gender-based violence, especially early childhood violence and abuse, intimate partner violence, and/or broader community violence, such as gang-related violence. Such social isolation was further exacerbated in destination settings by experiences of family separation and sex work-related stigma. Internalized stigma was common in participants’ narratives (i.e., feelings of guilt, embarrassment, and shame related to their perceived engagement in “immoral” or “dishonest” work) and was found to intensify such isolation by causing women to distance themselves from support networks (e.g., kinship, co-workers) in an effort to conceal their sex work involvement from family and friends:

I decided to come as far as I could, where nobody or my family could see me (when) I started working in this [sex work] (Carmen, 26 years old, 7 months in Tecún Umán).

Additionally, most women described experiencing distress, sadness, and anxiety, which they attributed to the intersecting influences of social isolation, stigma, fear and marginalization that they faced. These feelings were often linked to family separation–particularly the trauma and sadness associated with separation from children–as well as fear over what could happen to them during their migration process and border crossing. Unlike other difficulties described that tended to fade over time due to women’s resilience to adapt to their new work and environment, the stress caused by family separation prevailed over the course of migration for most women:

When I’m by myself, I start to cry. I miss my children and my family, and I wonder, oh God, why am I here? Why was my destiny this way? But if I stay in my country, where can I work? How can I maintain my children? How can I help them move forward? (Natalia, 38 years old, 6 years in Tapachula).

Women often faced increased difficulties related to factors linked to international migration, such as pronounced social isolation and deportation. Most women had migrated either alone or with a friend, and upon arrival to destination settings, most lacked social contacts beyond the person with whom they traveled. Social isolation following arrival was particularly enhanced for younger and unaccompanied migrants, and women who had migrated due to involuntary circumstances, such as when they were deported. As the account of a participant who had been deported demonstrated, women traveling alone and who had been deported faced increased vulnerability to social isolation and violence during transit and upon arrival to their country of repatriation:

They leave you there at the border, alone and helpless [a la ley de Cristo] without any money or anything, just hitchhiking, asking for a ride from a truck driver, a lift to our country (Alejandra, 25 years old, 7 months in Tecún Umán).

Most women participating in the study entered sex work following migration–a decision that was closely linked to economic needs and barriers to formal labour market opportunities experienced upon migration, including social isolation and stigma related to racialization and migration status. As the following participant explained, stigmatizing and discriminatory treatment based on international migration status was often perceived to exacerbate social isolation and to negatively affect access to other economic opportunities:

If you want to eat here, you must get sexually involved with a man, don’t expect a job in a house or in a clothing store, never expect that. Because as immigrants, we’re already looked upon as thieves because we all pay for what a few do wrong. They see a foreigner and they see us from head to toe, they’re afraid and they close the doors in our face (Gabriela, 28 years old, 16 years in Tapachula).

Peer support was described by many women to be an important means for negotiating the challenges of social isolation faced particularly within the context of recent migration and sex work entry. As duration lengthened in destination settings, many women were increasingly able to counteract the effects of social isolation by accessing peer support networks, which fostered safe and non-judgmental communication channels to express their feelings, concerns, and experiences, and to exchange advice and support:

I told her [a friend] that I didn’t know what to do… sometimes I felt like killing myself.

“No,” she would tell me,” if you kill yourself what is going to happen to your kids? You need to fight, you need to keep going” (Jimena, 35 years old, 8 years in Tapachula).

Participants developed relationships and support networks that to some extent attenuated the effects of social isolation with increased duration in their migration destination as well as experience in the sex industry. When women were able to build and access supportive relationships with other sex workers, the support this offered was valued highly, with many noting that they preferred talking to friends rather than counselors or psychologists. In some cases, women looking for solutions for one another’s needs would go as far as reaching out to other sex workers’ families for support if one of them was in trouble (e.g., struggling with addiction), exemplifying the long-lasting relationships of mutual help some women cultivated:

She [friend] became addicted [to heroin] and we [other sex workers] went to take her to a rehabilitation clinic because she was getting lost. Since I knew her mom, I called her mom and I told her: “Your daughter is getting lost in this addiction to drugs and alcohol” (…) (Now) she gives me advice too. I go to her house and she advises me: “the same way you advised me at one point, I advise you, leave that life…You got me out of my drug addiction and look, now I live happily and I want you to live the same way” (Marlen, 27 years old, 8 years in Tecún Umán).

### Peer support for addressing gaps in HIV/STI knowledge, prevention and resources

Peer support was an important component of HIV/STI prevention, particularly during the initial stages of arrival and sex work entry, when limited access to HIV and STI knowledge and prevention was most pronounced for most participants. In light of the limited availability of sex worker-friendly HIV/STI prevention services locally and the pervasive barriers women often faced when seeking such care, international migrant sex workers’ accounts indicated that peers often represented their main source of information about HIV and STI knowledge and prevention.

My peers told me that I needed to protect myself. They said I should do everything with a condom. They told me everything from their experience, so I wasn’t left with any [question] (Guadalupe, 29 years old, 2 years in Tapachula).

In addition to a lack of access to appropriate and timely HIV/STI prevention information and skills (e.g., condom demonstrations), economic challenges faced during recent arrival and sex work entry increased women´s vulnerability to HIV/STI acquisition, such as through clients´ offers of increased pay for unprotected sex. The following participant highlighted how her experiences as a newcomer rendered her particularly vulnerable to HIV and STIs within the context of work:

I only knew about AIDS, just what I had heard in comments and when that happened to me, that the condom broke, I would cry a lot because I was scared that I would have that sickness. And I couldn’t put them [condoms] on when I first started, maybe I put it on wrong (Victoria, 30 years old, <1 month in Tecún Umán).

Peer support related to HIV/STI prevention varied by work environment and to some extent, by country of work. Sex workers within formal indoor venues (e.g., bars, cantinas)–who primarily worked on the Guatemalan side of the border–frequently supported each other to access HIV/STI testing services by going to appointments together or by sharing information about services available. This type of support was often complemented by managerial support and/or by public health regulations and practices reinforcing periodical testing and scheduled regular visits to clinics, such as free-of-charge screenings. Women often valued the services and information they received through these channels:

They [bar managers] send us to the health services every Tuesday, and they give us [family] planning workshops and workshops on how to use condoms. “Don’t use Vaseline; don’t use lotion, because that warms it up, use a water based lubricant,” they say. They explain that we always have to use condoms because we could get an unwanted pregnancy, aside from [sexually transmitted] infections (Alejandra, 25 years old, 7 months in Tecún Umán).

On the other hand, women working on the street or in informal venues (e.g., hotels, parks), especially in Mexico, usually faced increased difficulties in supporting each other in HIV/STI knowledge and prevention due to the isolated nature of their work, and confronted greater gaps in HIV/ STI information, prevention and testing due to limited access to regular screening within informal settings. All participants who reported having an STI (n = 4) worked in informal settings. Sex workers outside of formal establishments reported further limitations to HIV/STI prevention, such as enhanced violence and harassment from authorities when carrying condoms, in comparison with their peers working in more supportive spaces. Women working in informal settings also faced increased violence from clients during condom negotiation due to the lack of safety measures and peer support often present within indoor venues, as the account of a woman who exchanged sex in a trailer demonstrated:

The other time a client was killing me, he hurt me, he twisted my arm. He took the condom off and he took my hands and he grabbed me like an animal! He hurt me a lot! I came out crying, I wanted to die! (Concepción, 39 years old, 9 years in Tapachula).

These enhanced vulnerabilities made peer support even more vital for this group. It also played an important role in supporting HIV/STI prevention during the first stages of sex work entry, with workers often providing guidance and advice to each other regarding the consistent use of condoms and strategies for dealing with difficult or uncooperative clients. Indeed, most participants working in indoor venues and some women working in informal settings said that they learnt the need to protect themselves through other sex workers:

The peers tell you that you have to protect yourself because they see that it’s your first time and they see you are clumsy; they explain that you have to use a condom (and) what you must not do. In every place you go there is always someone good (Bertha, 35 years old, 2 years in Tecún Umán).

(There is a dude (who) doesn’t use a condom, he pays $400 pesos but he never tells us what disease he has. I told the girl [another sex worker]: “this man has AIDS, don’t get involved with him (…) I said that to her and many others did: “You’re not going to recover your health or your life with $400 pesos” (Gabriela, 28 years old, 16 years in Tapachula).

In addition, they provided each other emotional support when they were worried about health issues, and took care of each other when they were sick. Women also described sharing condoms or lubricants when needed and lent each other money to pay for medicines and other health-related fees (e.g., clinic visits). However, limitations on the types of information and support that international migrant sex workers received from each other in regard to HIV/STI prevention were also identified. Although many women had friends emphasize the importance of wearing a condom or refusing client offers for unprotected sex, only a small minority had received instruction on how to properly use condoms. In addition, some had only been able to access this information after they had already started doing sex work, which for many led to having had unprotected sex with their first clients prior to their exposure to HIV/STI prevention methods from peers.

### Workplace violence and peer support for enhancing safety

International migrant sex workers described violence within their day-to-day work environments as very common, which was perpetrated by multiple actors, primarily clients and government authorities such as police, health inspectors, and immigration agents. Although peers offered each other support to mitigate, escape from, or cope with such violence, fear of repercussions related to migration and criminalization, such as increased violence from authorities or deportation, limited the type and extent of assistance that peers could provide in this respect.

### Violence from clients

Physical, psychological and sexual violence from clients occurred throughout all stages of sex work and migration, and was described as most likely in the context of clients’ substance use and during condom negotiation. Vulnerability to violence was also greatly shaped by work environment. While women working in indoor venues often reported protection from managers and staff, women working at outdoors or in informal venues seldom felt protected by hotel staff and described increased feelings of danger:

I'm scared in the street because there are many evil men and no one is responsible for you. At a business [formal establishment] you have the owners or security guards who can defend you at a given time. If there is any problem, they help us; in the street we have to manage ourselves as we can (Sonia, 37 years old, 12 years in Quetzaltenango).

Sex workers discussed the different mechanisms of peer support they often developed to mitigate and support each other in the face of such violence, although these mechanisms were generally employed and accessed by workers over time, and were usually less available to newcomers and more socially isolated workers. These protective strategies included writing down clients’ license plates, checking in on each other during dates, and advising each other not to service intoxicated clients or clients with a reputation for violent behaviour:

If one of us leaves in a car, any of our peers writes down the license plates. If later on we see that she hasn’t come back, we call her on her cell phone and if she doesn’t answer, we go look for her. We always have credit on our cell phone for anything that could happen (Natalia, 38 years old, 6 years in Tapachula).

When women experienced violence from clients, they also offered each other emotional and practical support by listening to each other and providing advice:

There was another young woman who was always at the park, she saw me coming back crying. She asked me what had happened to me. And I began to tell her everything (about an experience of violence from a client). She said that I shouldn’t have gone. I told her that my son was sick and that I needed the money. She said: “look, the good thing is that he didn’t kill you” (Luciana, 20 years old, 19 years in Tapachula).

Despite the supportive effects of peer advice and safety supports in the face of workplace violence, international migrant sex workers often described serious limitations to their agency in reporting such violence due to fear of negative repercussions or retribution by authorities, such as increased violence, deportation, or imprisonment. As one worker explained her experience of having been threatened by a government official:

He [officer from the government] would say: “if you plan on pressing charges, we are the ones who are in charge here, we will send you to your country and I won’t let you be able to come back to this country” (Alejandra, 25 years old, 7 months in Tecún Umán).

### Violence from authorities

Participants commonly faced harassment, violence, and other human rights violations perpetrated by immigration authorities, police, and public health inspectors (i.e. public health authorities in charge of the supervision of mandatory HIV/STI testing compliance among sex workers) [7,9], whose roles in the enforcement of public health regulations surrounding sex work, immigration, and sex work criminalization were often blurred [6]. Migrant sex workers were particularly vulnerable to violence by authorities within the context of circular mobility to their countries of origin, as well as during the process of deportation, to which almost two-thirds of participants had been subject at least once.

Given that authorities were, in many cases, a source of violence instead of a source of protection, migrant sex workers described various peer support mechanisms developed to protect each other from abuses by authorities. For example, women developed means of alerting one another when authorities were coming so that they could hide or leave the premises and, when necessary, peers sometimes lent each other money to pay off bribes or get out of jail:

When the [police] patrol comes from afar, (some) of the girls (are) at the corner and they say that they [police officers] are coming, so we hide…Once we all put money to pay for one of the girls, we told her: “we are going to get you out [from jail] and when you are good to go, then you’ll pay us” (Cecilia, 38 years old, 3 years in Tapachula).

### Variations and limitations in peer support

Variations in access to peer support were frequently noted among migrant sex workers, usually related to the stage of migration, sex work entry, and location or work environment. Peer support was most crucial during the first stages of migration and sex work entry, when participants described the greatest need for guidance and advice due to pronounced experiences of social isolation, stigma, violence, and lack of access to HIV/STI knowledge and prevention.

Peer support was more prevalent in indoor formal venues and more likely to be reported on the Guatemalan side of the border, where some establishments encouraged peer support practices, such as sharing knowledge about HIV/STI prevention and going to the clinics together. Women in these settings reported accessing to HIV/STI screening more often than women in Mexico working at informal venues, where peer support occurred more organically.

In the latter cases, peer support was particularly important in regard to safety and protection from violence by clients and authorities, as women working in informal venues worked independently and did not have the protection and support from managers that often existed in formal venues. Women working at the street or at informal venues also faced increased barriers to peer support, such as those associated with frequent mobility and constantly changing one’s workplace to avoid police harassment, which diminished the support that they could give to each other and often resulted in displacement from peers.

Limitations to international migrant sex workers’ agency to offer and access meaningful support from each other were also identified in relation to broader structural circumstances surrounding migration and sex work environments. As many participants travelled between their country of destination and origin regularly, this frequent mobility and circular migration difficulted nourishing long-lasting relationships and forming established social organization or mobilization. Additionally, the type of peer support that migrant sex workers could offer each other against violence and human rights violations by clients and authorities was limited. While some of the health and socioeconomic challenges women faced tended to decrease over time, violence prevailed throughout their sex work and migration experiences. Hence, existing power imbalances placed important limitations on the collective agency that migrant sex workers could have in altering the intersecting social factors that increased vulnerability to violence and HIV/STI.

Tension and competition among workers also posed barriers to peer support for women within some work environments. In some cases, international migrant sex workers who had not disclosed their sex work to their families and friends described their fear of gossip by other sex workers from their community of origin as a reason for avoiding building relationships with other sex workers, dreading the consequences this could present if their sex work status was revealed to their contacts in their home countries. In some cases, competition between workers caused women to consider peers as more of a threat than a source of support. This competition was more present within street-based settings than formal indoor venues, and was particularly prevalent during initial sex work entry. As one worker explained:

I was a newcomer and I made a lot of money and they [other sex workers] didn’t. They tried to hit me and all that…They kicked me out, they said that the street was theirs and used to head home (Pia, 20 years old, 3 years in Tapachula).

Participants found different ways to cope with these tensions, such as working on different schedules than other women or having friends negotiate with other sex workers on their behalf:

When you are new, sometimes, the girls that already work here don’t like it. She [other sex worker] knew them all because she has been working here for a while. She talked with the ones that have more time working here and she told them, so I was able to work without problem (Rita, 44 years, 2 years in Tapachula).

Finally, women sometimes described episodes of violence or robbery among sex workers themselves. These were more prevalent during recent arrival and sex work entry and usually targeted younger women, who learnt how to negotiate with these difficulties over time:

I was a newcomer and I was clueless. She [other sex worker] took me out for a drink but because I didn’t know how to drink, I got drunk immediately and once she saw I was drunk, she took the money away from me. But I remembered that I had money. The next day I went to tell her. She made a big deal out of it and she told me that she was not going to return anything. “Fine”, I said, “keep my money”. Nowadays when I see her, I don’t talk to her (Luciana, 20 years old, 19 years in Tapachula).

## Discussion

Peer support was found to be an important mitigating factor for some of the challenges faced by migrant sex workers, particularly in relation to experiences of social isolation and stigma, HIV/STI knowledge and prevention, and violence within the workplace. Interpersonal, institutional and structural challenges to accessing peer support were identified, including difficulties establishing long-lasting relationships and other forms of social participation due to frequent mobility, as well as tensions and competition between peers within some work environments. Variations in access to peer support were frequently noted among international migrant sex workers both in Mexico and Guatemala. Similar to research findings from Canada, we found peer support in these settings to be more crucial during the earlier stages of migration (e.g., recent arrivals within the last year) and among those with less sex work experience, and tended to be more present in indoor formal venues where workers’ ability to work collectively as well as managerial policies and practices often supported its development [12,34].

Our results are also supported by previous research conducted across diverse locations including Canada, India, Brazil, and the Dominican Republic, documenting positive health outcomes resulting from community empowerment-based approaches and health interventions focusing on mobilizing communities and promoting social cohesion and access to peer-based supports among sex workers [34,37,38,40,65]; and by other studies conducted in settings such as Mexico and Zimbabwe, which found that both peer support and competition are present among sex workers [41,66].

Our findings build on this work by adding a migrant-specific lens, thus contributing to the understanding of the unique characteristics of peer support among migrant populations, who often face increased violence and harassment from authorities, and pronounced social isolation due to constant mobility, fear of incarceration or deportation, and dislocation from previous support networks. This lens also contributes to the understanding of the implications of peer support in the implementation of community empowered-based approaches and health interventions among migrant and mobile communities aiming to improve the community factors determining women’s health and wellbeing.

Our findings indicate that peer-led and community empowerment interventions represent a promising strategy for promoting the health, safety and human rights of migrant sex workers in Central America and potentially elsewhere–for example, such interventions may support women’s capacity to gain improved control over their working conditions and over the diverse and layered challenges they face in the context of migration and sex work status, such as those related to sexual health and safety [67].

Our results demonstrate that interventions addressing HIV/STI prevention, violence, and other health and social inequities among migrant sex workers should highlight and build upon their resilience and individual and collective agency; and should be tailored to the unique needs faced by migrant women. For example, as migrant sex workers often travel on a regular basis and many face mobility-related barriers to health access, health interventions could consider incorporating mobile health (mHealth) or other communication technologies as a means of sharing information and supporting remote access to different types of peer and health-related supports in transnational contexts [68,69]. This may be particularly appropriate given that some migrant sex workers already report reliance on mobile technologies to access information about health and safety from their peers, healthcare providers, and other sources. The types of support that could be facilitated through mHealth strategies could include appropriate and timely information about health, working conditions, and clients provided by peers, group chats with peers, and/or emergency or health care telephone services, to name a few [70,71]. In addition, the use of technology could contribute to increased social cohesion and mobilization among international migrant sex workers by forging connections among otherwise isolated women [37]. Although participatory and community empowerment-based approaches and increased social cohesion have been linked to reductions in stigma, violence, and ultimately, HIV/STI-related risks amongst sex workers in other contexts [34,37–39], to our knowledge no previous research or interventions have addressed this critical aspect of health and wellbeing amongst migrant women engaged in sex work.

Other suggested peer-based intervention strategies to promote the health and wellbeing of international migrant sex workers could include community/peer engagement to raise awareness about legal and human rights issues, HIV and STI prevention, and safety within the workplace; establishment of community-led drop in centers for peer collaboration, particularly in relation to guidance with HIV and STI prevention and mental health support; and broader peer-based outreach and human rights advocacy efforts, especially in informal and outdoor venues [67]. Such interventions should be developed in collaboration with–and ideally, leadership by–migrant sex workers and local organizations advocating for the rights of women, sex workers, and/or migrants [72]. While international migrant sex workers require support at all migration and sex work stages, interventions that focus on the initial stages of migration and sex work entry are initially recommended, as the narratives of participants suggested that this was a particularly pronounced time of vulnerability and social isolation in which supportive interventions would be most urgently needed.

However, while peer support and peer-led and community empowerment interventions can serve to mitigate some of the challenges faced by migrant sex workers, these are not sufficient by themselves. Peer-led and community empowerment interventions with migrant sex workers should be complemented by broader public policy and structural changes–such as decriminalization of sex work, improved access to safer work environments, and reduction of stigma related to sex work and migration status–to more comprehensively support the health, safety, and rights of migrant sex workers [5,8,73]. Thus, there remains a critical need for research facilitating dialogue, participation, cooperation, and collective mobilization among migrant sex workers geared towards the identification of the multi-level factors that constrain and/or promote health [11,13,14], and the creation of collaborative spaces to promote agency and contest these forces[37,74], particularly within more underserved and less well-researched contexts characterized by intense violence and gender-based human rights violations, such as Central America.

### Strengths and limitations of the study

Challenges accessing more hidden and criminalized populations, together with migrant sex workers’ unique concerns regarding research participation (e.g., stigma, legal concerns) [3] could have resulted in an under-representation of more marginalized migrant sex workers in this study. To abate these difficulties, we employed unobtrusive recruitment mechanisms across different indoor and outdoor settings in Mexico and Guatemala, worked in close collaboration with local organizations, and built and strengthened rapport with participants through prolonged processes of informed consent and long-term collaboration with community partners. In addition, we asked questions about both individual and group experiences in the past and present, obtaining important insights about peer support, migration and sex work.

Finally, recognizing the impact of stigma, researcher roles, and social desirability bias on participants’ potential willingness to discuss experiences perceived as stigmatizing, we developed explicit strategies to address this, including working closely with our community partners who maintain long-term trusting relationships in the community, and developing explicit training and research protocols to protect confidentiality and create an open, safe, reciprocal, and non-stigmatizing interview atmosphere [73].

## Conclusions

Despite variations and limitations to peer support and to the individual and collective agency of international migrant sex workers, peer support was important for combating social isolation, supporting access to HIV and STI knowledge and prevention, and mitigating and coping with violence within the workplace, particularly for recent international migrants new to the sex industry. Results point towards the need for peer-led community empowerment health interventions tailored to the needs of migrant communities alongside broader public policy and structural changes to support sex workers’ rights and health. Future peer support and community mobilization interventions should be further explored and tailored to the contexts of migrant women; such studies should also consider the potential for various communications strategies (e.g., cell phones) for reducing isolation and supporting the health and human rights of migrant sex workers in Latin America and elsewhere.
